# Valve-Sparing Aortic Root Replacement as First-Choice Strategy in Acute Type a Aortic Dissection

**DOI:** 10.3389/fsurg.2019.00046

**Published:** 2019-08-06

**Authors:** Hug Aubin, Payam Akhyari, Philipp Rellecke, Christina Pawlitza, George Petrov, Artur Lichtenberg, Hiroyuki Kamiya

**Affiliations:** ^1^Department of Cardiovascular Surgery, University Hospital Düsseldorf, Heinrich-Heine University Düsseldorf, Düsseldorf, Germany; ^2^Department of Cardiac Surgery, Asahikawa Medical University, Asahikawa, Japan

**Keywords:** acute aortic dissection type A, valve-sparing aortic root replacement, David procedure, treatment strategy for aortic dissection, aortic valve repair, supracoronary replacement of the ascending aorta

## Abstract

**Background:** Although, in theory, valve-sparing aortic root replacement (*David procedure*) is an ideal surgical option for acute aortic dissection type A (AADA) it is usually not regarded as the first-choice treatment due to the emergency setting and the relative complexity of the procedure. Here, we report the results of a consecutive, single-surgeon series of 45 AADA patients with the *David procedure* as first-choice treatment strategy.

**Methods and Results:** Between September 2009 and July 2013 a total of 49 patients with AADA were consecutively operated by the same surgeon at our institution. The *David procedure* was the treatment of choice for the proximal aorta unless aortic valve pathology or critical preoperative patient status advocated against it. Median follow-up was 5.0 years (CI95%, 4.0–6.0). Out of the 45 patients included in this study the *David procedure* was performed in 28 patients (62.2%), while in 17 patients (37.8%) an alternative surgical strategy had to be pursued. Although X-clamping (168.5 ± 41.7 vs. 110.3 ± 51.1 min; *p* = 0.001), cardiopulmonary bypass (CPB) (245.0 ± 62.4 vs. 211.8 ± 123 min; *p* = 0.029) and total operation time (383.8 ± 88.5 vs. 312.8 ± 144.8; *p* = 0.047) were significantly longer in the David-group as compared to the non-David group, there was no difference in major complication rate as well as 30-day (17.9 vs. 23.5%; *p* = 0.645) and 5-year mortality (28.6 vs. 35.3%) between groups.

**Conclusions:** This small series indicates that the *David procedure* may be safe and feasible as a primary surgical treatment strategy for AADA.

## Introduction

Acute aortic dissection type A (AADA) is a life-threatening condition with an extremely high mortality rate, in which immediate surgical intervention is mandatory (1, 2). The primary goal of surgical treatment for AADA is the resection of the dissection entry site in order to exclude the false lumen from the systemic blood flow and to prevent further dissection or fatal rupture of the aorta. Depending on the affection of the aortic root, supracoronary replacement of the ascending aorta (SCR) or composite replacement of the aortic root and ascending aorta (CVR), also referred to as the *Bentall procedure*, are the standard surgical procedures in the treatment of AADA. Due to concomitant aortic arch pathology, those techniques are usually performed with an open distal anastomosis and under hypothermic circulatory arrest (3).

In the last decade, there has been increasing experience with the valve-sparing aortic root replacement technique (re-implantation technique, *David procedure*) for operative treatment of AADA with encouraging results (4–8). Theoretically, the *David procedure* is an ideal surgical option for AADA with dissected aortic root and unimpaired valvular cusps, since it completely removes the diseased aortic tissue with very low risk of subsequent late aortic root complications such as re-dissection, pseudo aneurysms of the ascending aorta or aortic valve insufficiency—otherwise common AADA post-treatment complications (9, 10). However, the *David procedure* is a complex and technically demanding operation, usually associated with prolonged operation times as compared to non-valve sparing alternatives. Also, clear patient selection criteria for the use of the *David procedure* in AADA has not been well-reported yet, due to extremely varying preoperative patient status, complex and heterogeneous dissection pathologies as well as differing individual surgeon strategies, which impede standardization. Therefore, it still remains questionable whether in an emergency setting the *David procedure* might be used as first choice treatment strategy for AADA or whether patients might not benefit from a less complex approach.

Here, we report the results of a single-surgeon series of 45 consecutive AADA patients in whom the *David procedure* was employed as first choice treatment strategy following a clear treatment algorithm, hypothesizing that with adequate patient selection a valve-sparing aortic root replacement technique is a valid first choice treatment strategy for AADA patients despite increased technical complexity of the operation itself.

## Patients and Methods

### Patient Cohort

From September 2009 until July 2013, 49 consecutive patients with AADA underwent emergency surgical treatment by a single surgeon at the Düsseldorf Heart Center. Aortic dissection was considered as an AADA if symptoms occurred within 14 days and dissection of the ascending aorta was confirmed by computed tomography scans, magnetic resonance imaging, and/or transesophageal echocardiography (TEE). The majority of patients were out-of-center patients diagnosed in external hospitals and transferred to our institution for emergency surgery. If AADA diagnosis was clear at admittance, patients were directly transferred to the operation room for immediate surgery. In all other cases diagnosis was completed at our institution prior to operation. Out of the 49 patients, three patients with previous cardiac surgery and one patient with intraoperative AADA during non-aortic primary cardiac procedure were excluded from the following further analyses, thus a total of 45 emergency AADA patients were included into this study.

### Decision Criteria for Surgical Strategy

**Figure 1:**
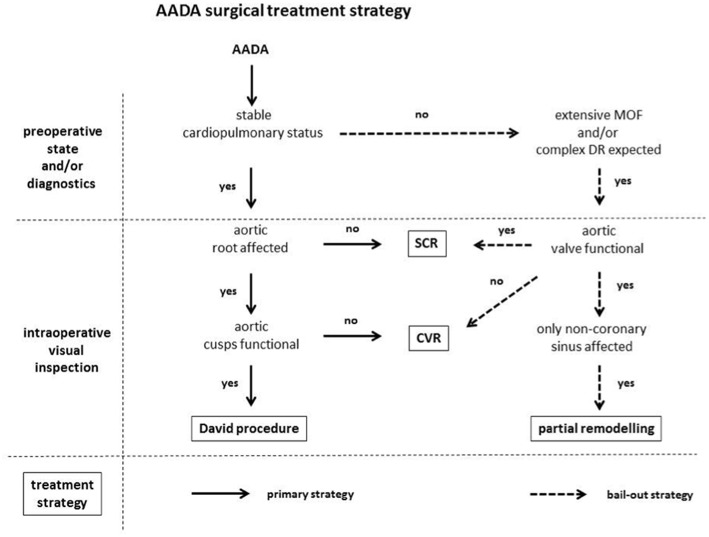
AADA surgical treatment strategy. AADA, acute aortic dissection type A; MOF, multi-organ failure; DR, distal repair; SCR, supracoronary replacement; CVR, combined valve and aortic replacement.

In this series of 45 consecutive AADA patients the *David procedure* was the treatment of choice for the proximal aorta unless aortic valve pathology or critical preoperative patient status advocated against it. Therefore, decision for a valve-sparing approach was undertaken independently from preoperative echocardiographic aortic valve assessment and degree of regurgitation. Feasibility of the *David procedure* was determined solely intraoperative after visual inspection of dissection pathology including the aortic root and valvular cusp morphology, with additional consideration of the patient's preoperative clinical status and diagnostic findings. Decision criteria and reasons for deviation from the *David procedure* as primary treatment procedure are highlighted in the treatment algorithm in Figure 1. Briefly, all dissections involving the aortic root with functional aortic cusps underwent the *David procedure* regardless of concomitance of aortic regurgitation. Only under one or more of the following circumstances a less complex surgical technique, such as SCR or CVR, was employed independent from cusp morphology as bail-out strategy in order to keep operation time as short as possible: (1) very instable cardiopulmonary status with CPR prior to skin incision; (2) preoperatively manifest extensive multi-organ failure due to malperfusion; and/or (3) preoperative CT-finding suggesting that a complex distal repair in the region of the aortic arch was likely. In patients with stable cardiopulmonary status and dissections affecting only the non-coronary sinus of Valsalva with high probalities of a complex distal repair a partial aortic root remodeling technique in form of *1/3 Yacoub procedure* (*partial remodeling*) was performed. If aortic valvular cusp morphology advocated against a valve sparing technique as intraoperative judged by the primary surgeon a CVR was applied.

### Surgical Technique

The consistent strategy of the employed treatment strategy was the *in toto* resection of the pathological proximal aortic wall without the use of any biological glue whenever possible. Therefore, the *David procedure* was performed with a straight tubular graft size-adapted to the optimal diameter of the aortic root (David I). For the distal graft anastomosis, all operations were performed under hypothermic circulatory arrest with antegrade selective cerebral perfusion to allow open anastomosis. Treatment strategy included a *total arch replacement* whenever a tear could be identified in the aortic arch, performed with a four-branched vascular prosthesis in all patients except for two patients early in the series. In those two patients, the aortic arch was transected between the brachiocepalic and the left common carotid artery with separate reconstruction of the brachiocephalic artery, which was defined as a *partial arch replacement*. A *total arch replacement* was also performed in two patients without a clear presence of a tear in the aortic arch, but because of the relative young age of the patients and in order to prevent late complications in this region. If the aortic arch was free of tears, then it was transected obliquely from the origin of the brachiocephalic artery down to the distal arch and anastomosed with the vascular graft leaving the supraaortic vessels *in situ*, this was defined as *proximal arch replacement*.

Cannulation site was determined according to preoperative diagnostic imaging. Briefly, the right axillary artery was first choice for arterial cannulation if free of dissection. Alternatives for arterial cannulation were, the ascending aorta *via* Seldinger-technique, or one of the femoral arteries if not affected by the dissection. Continuous monitoring of the cerebral blood oxygen saturation was routinely performed. Cardioplegic solution was given in antegrade fashion at the beginning of the series, however, to the end of the series it was applied in retrograde fashion through the coronary sinus to avoid injury of potentially fragile coronary ostia. In cases of retrograde cardioplegia, the superior vena cava and the inferior vena cava were cannulated separately and a cannula for retrograde cardioplegia was inserted into the coronary sinus under direct vision. In cases of weaning difficulties from the cardiopulmonary bypass and/or impaired regional wall contractility in TEE additional coronary artery bypass grafting (CABG) was done—if necessary—in beating heart technique under cardiopulmonary bypass (CPB).

### Data Management

All data relevant to analysis of clinical outcome including 30-day mortality and later midterm follow-up were prospectively collected and entered into an institutional data registry. Analysis comprised retrospective analysis of early outcome and additional follow-up data which consisted of telephone contact of the patients, patient relatives and/or primary general physicians. Each surviving patient was invited to an echocardiographic examination at our institution, performed by one single examiner following institutional standards. Thirty-day follow-up was a 100% complete, mid-term follow-up 89%. An additional mid-term echocardiographic exam could be performed in 85% of the surviving patients who had received a *David procedure*.

### Statistical Analysis

For analysis, patients were divided into 2 subgroups as determined by the applied surgical treatment: patients who underwent the *David procedure* (David-group) and patients who received an alternative non-David operation (non-David group). These groups were analyzed and statistically compared with each other. Results are expressed as mean ± standard deviation. Statistical analysis was performed using Student's *t*-test for continuous variables or χ^2^ tests (Fisher's exact tests if *n* < 5) for categorical variables. A *p*-value < 0.05 was considered significant. All statistical analyses were performed using SPSS 16.0 software (SPSS Inc., Chicago, IL).

## Results

### Patient Characteristics and Preoperative Status

Out of the 45 patients included in this study the *David procedure* was performed in 28 patients (62.2%), while in 17 patients (37.8%) a different surgical strategy had to be pursued, following the treatment algorithm outlined in Figure 1. Patient characteristics including demographic data and preoperative clinical status are outlined in Table 1. Although, there were no significant differences between both groups, the non-David-group had a tendency to increased preoperative morbidity with an increased incidence of cardiac tamponade (35.5 vs. 17.9%; *p* = 0.187), CPR (5.9 vs. 0%; *p* = 0.378), (35.3 vs. 14.3%; *p* = 0.100) as well as limb ischemia (29.4 vs. 14.3%; *p* = 0.198) as compared to the David-group, respectively (Table 1). Further, there was no difference in the incidence and severity of echocardiographic preoperative aortic regurgitation between both groups (AR severity: 2.1 ± 1.2 vs. 2.3 ± 1.3; *p* = 0.554; David-group vs. non-David-group, respectively) (Table 1).

**Table 1:** Patient characteristics.

	**David-group** **(*n* = 28)**	**Non-David group** **(*n* = 17)**	p^*^
Age (years)	60.8	63.2	0.460
Male	16 (57.1%)	11 (64.7%)	0.615
Emergency operation	28 (100%)	17 (100%)	n.a.
Cardiac tamponade	5 (17.9%)	6 (35.3%)	0.187
Neurological dysfunction	4 (14.3%)	6 (35.3%)	0.100
Visceral ischemia	2 (7.1%)	1 (5.9%)	0.684
Limb ischemia	4 (14.3%)	5 (29.4%)	0.198
Initiation under CPR	0	1 (5.9%)	0.378
AR severity	2.1 ± 1.2	2.3 ± 1.3	0.554

*CPR, cardiopulmonary resuscitation; AR, aortic valve regurgitation. Data presented as mean ± standard deviation. Bold values indicate statistical significance (p < 0.05)*.

* *Student's t-test for continuous variables; χ^2^ tests for categorical variables*.

### Surgical Approach and Intraoperative Data

The specific surgical approach and intraoperative procedural data are outlined in Table 2. In the non-David group, out of a total of 17 patients, 10 patients underwent SCR (58.8%), 3 patients CVR (17.6%), and 4 patients the *partial remodeling* (23.5%) (Table 2). Operation modality for the distal site was homogenously distributed between both groups, with *proximal arch replacement* in 64.3 vs. 64.7%, *partial arch replacement* in 3.6 vs. 5.9% and *total arch replacement* in 32.1 vs. 29.4% of the cases (*p* = 0.926; David-group vs. non-David-group, respectively) (Table 2). Additional concomitant coronary bypass grafting was necessary in 5 patients of each group (17.9 vs. 29.4%; *p* = 0.366; David-group vs. non-David-group, respectively) with target vessels listed in Table 2. After valve-sparing aortic root replacement there was no relevant intraoperative aortic valve malfunction as evaluated by intraoperative TEE; intraoperative correction of primary valve reconstruction was 0% in the David-group (Table 2).

**Table 2:** Intraoperative procedural characteristics.

	**David-group** **(*n* = 28)**	**Non-David group** **(*n* = 17)**	p^*^
Operation for proximal site			
David procedure	28		
SCR		10	
CVR		3	
Partial remodeling		4	
Operation for distal site			0.926
Prox. arch replacement	18 (64.3%)	11 (64.7%)	
Partial arch replacement	1 (3.6%)	1 (5.9%)	
Total arch replacement	9 (32.1%)	5 (29.4%)	
Cannulation site			0.751
RAA	10	5	
AA	16	8	
FA	2	4	
CABG	5 (17.9%)	5 (29.4%)	0.366
LAD	3	1	
RCX	4	0	
RCA	5	5	
Operation time (min)	383.8 ± 88.5	312.8 ± 144.8	**0.047**
CPB time (min)	245.0 ± 62.4	211.8 ± 123.2	**0.029**
X-clamping time (min)	168.5 ± 41.7	110.3 ± 51.1	**0.001**
HCA time (min)	37.9 ± 29.6	40.1 ± 28.8	0.828
SACP time (min)	33.7 ± 27.2	36.4 ± 23.7	0.745
Lowest temperature (°C)	25.4 ± 1.0	26.0 ± 1.2	0.821
Intraoperative correction of primary valve reconstruction	0 (0%)	n.a.	

*SCR, supracoronary ascending aorta replacement; CVR, composed aortic valve and aorta ascendens replacement; RAA, right axillary artery, AA, ascending aorta; FA, femoral artery; CPB, cardiopulmonary bypass; HCA, hypothermic circulatory arrest; SACP, antegrade selective cerebral perfusion; CABG, coronary artery bypass grafting; LAD, left anterior descending artery; RCX, circumflex artery; RCA, right coronary artery; X-clamping time, duration of aortic cross clamping. Data presented as mean ± standard deviation. Bold values indicate statistical significance (p < 0.05)*.

* *Student's t-test for continuous variables; χ^2^ tests for categorical variables*.

Although circulatory arrest time, selective cerebral perfusion time and lowest core body temperature did not differ between groups, aortic X-clamp times (168.5 ± 41.7 vs. 110.3 ± 51.1 min; *p* = 0.001), CPB times (245.0 ± 62.4 vs. 211.8 ± 123 min; *p* = 0.029) and total operation times (383.8 ± 88.5 vs. 312.8 ± 144.8; *p* = 0.047) were significantly longer in the David-group as compared to the non-David-group (Table 2). Detailed intraoperative procedural characteristics are listed in Table 2.

### 30-day Mortality and Postoperative Complications

There was no significant difference in 30-day mortality between both groups, with 17.9% mortality in the David-group and a slightly higher mortality of 23.5% in the non-David-group during the first 30 days (*p* = 0.645) (Table 3). Further, there was no significant difference in postoperative major complications between both groups, including stroke, visceral ischemia necessitating abdominal surgery re-exploration due to bleeding, low cardiac output syndrome or renal failure (Table 3). Detailed data on postoperative complications are listed in Table 3. Postoperative echocardiographic assessment of the reconstructed aortic valves revealed no significant aortic valve regurgitation in any of the patients who underwent the *David procedure*, with a mean degree of aortic valve regurgitation of 0.6 ± 0.7 (Table 3).

**Table 3:** Thirty days follow-up.

	**David-group** **(*n* = 28)**	**Non-David group** **(*n* = 17)**	p^*^
30 d-mortality	5 (17.9%)	4 (23.5%)	0.645
Stroke	3 (10.7%)	3 (17.6%)	0.407
Bowel resection due to visceral ischemia	4 (14.3%)	1 (5.9%)	0.365
Re-Thoracotomy due to bleeding	4 (14.3%)	4 (23.5%)	0.344
LCOS	1 (3.6%)	2 (11.8%)	0.316
Renal failure	4 (14.3%)	5 (29.4%)	0.198
AR severity	0.6 ± 0.7	n.a.	

*LCOS, low cardiac output syndrome; AR, aortic valve regurgitation; n.a., not available/applicable. Bold values indicate statistical significance (p < 0.05)*.

* *Student's t-test for continuous variables; χ^2^ tests for categorical variables*.

### 5-Year Mortality and Midterm Aortic Valve Function

**Figure 2:**
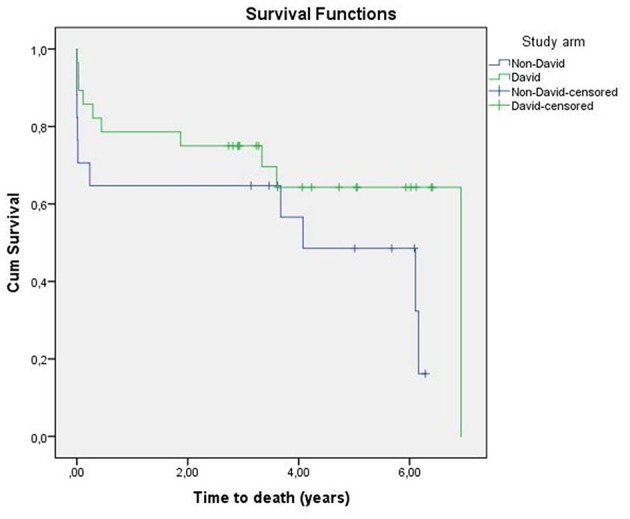
Kaplan-Meier survival David vs. non-David group.

After a median follow-up of 5.0 years (CI95%, 4.0–6.0) mortality in the David-group was 28.6 vs. 35.5% in the non-David-group (*p* = 0.136) (Table 4). Kaplan-Meier-survival of the study population is outlined in Figure 2. None of the patients in the David-Group had to be re-operated due to a primary operation related cause, while one patient in the non-David group did receive an aortic valve replacement after failed *partial remodeling*. Follow-up echocardiographic assessment of the reconstructed aortic valves in the David-group revealed a mean degree of aortic valve regurgitation of 0.6 ± 0.7, matching the early postoperative assessment.

**Table 4:** 5-year follow-up.

	**David-group** **(*n* = 28)**	**Non-David group** **(*n* = 17)**	p^*^
FU-Mortality	8 (28.6%)	6 (35.3%)	0.136
Need of Re-operation due to valvular or aortic cause	0 (0.0%)	1 (5.8%)	
AR severity	0.6 ± 0.7	n.a.	
Aortic valve orifice (cm^2^)	2.4 ± 0.3	n.a.	
Mean gradient (mmHg)	6.4 ± 3.1	n.a.	
V_max_ (m/s)	1.6 ± 0.3	n.a.	

*FU, follow-up; AR, aortic valve regurgitation; n.a., not available/applicable. Bold values indicate statistical significance (p < 0.05)*.

* *Student's t-test for continuous variables; χ^2^ tests for categorical variables*.

## Discussion

The optimal surgical treatment strategy in patients presenting with AADA is still subject of controversy. As the valve-sparing aortic root replacement procedure (*David procedure*) has shown to result in excellent long-term clinical outcomes (11). It is increasingly being employed in ADAA patients (4–8, 10–15). However, question remains whether less complex and more time-saving approaches might be of greater benefit for these high risk patients in an emergency setting. In this study, we analyzed a continuous series of 45 ADAA patients, in which 62.2% of the patients received a *David procedure* for surgical treatment of the distal aorta, demonstrating similar early and midterm outcomes to the rest of patients in whom an alternative strategy had to be pursued.

As illustrated by the German Registry for Acute Aortic Dissection Type A (GERAADA) reinforcement of the sinus Valsalva using biological glue is still the most frequent technique in the treatment of AADA (16, 17). Although SCR can undoubtedly be performed with lower technical complexity and shorter procedural times, concerns remain about using biological glue and leaving diseased aortic wall tissue *in situ*, leading to late complications associated with aortic root pathology (18). Potential re-dissection or late aneurysm formation may bear a high risk for the patient requiring further surgical treatment of the proximal ascending aorta. This is especially the case when aortic regurgitation due to aortic aneurysms develops with a reported incidence of 25% up to 45% (19). Different studies have reported a need of re-operation between 4.7 and 10.7% due to re-dissection after use of gelatin-resorcinol-formaldehyde (GRF) glue to reinforce the diseased aortic root (20, 21). Therefore, the risk of re-dissection and aneurysm formation with serious implications for the patients after use of biological glue to reinforce the diseased aortic wall should not be disregarded.

In addition to the risk of late aortic root pathology we would like to highlight the risk of early postoperative re-dissection and/or rupture of the aortic root in cases where the damaged aortic wall is not resected *in toto*, as was the case in two patients of our series. Although it cannot be ruled out that re-dissection and aortic root rupture originated from an initial technical problem, the risk of ongoing aortic pathology cannot be fully eliminated if diseased aortic tissue is left *in situ*. Hence, one of the major advantages of the *David procedure* lies in the *in toto* resection of the presumably pathologic aortic root.

Another advantage of the *David procedure* is the excellent hemostatic result that can be achieved on the proximal anastomosis site, due to the reinforcement with double suture lines on the aortic root. Although a focused analysis on postoperative bleeding could not be included in this study, the impression of enhanced hemostasis with the *David procedure* as compared to the non-David procedures is supported by a clear trend to reduced incidence of re-thoracotomy due to bleeding in the performed analysis (14.3 vs. 3.5%), which has also been seen in other comparable studies (8).

On the other hand, technical problems originating from the re-implantation of the coronary buttons may be regarded as a disadvantage of the *David procedure* over alternatives such as the SCR. Technical failures, e.g., kinking of the repositioned coronary artery occur frequently after re-implantation of the right coronary ostium requiring additional coronary artery bypass grafting. In the present study four patients in whom the coronary ostia were re-implanted (two underwent *David procedure* and two CVR) received an additional single bypass to the RCA, although it could not be elucidated if the cause lied in a pre-existing coronary artery disease or due to technical issues.

Despite the theoretical advantages of the David procedure in AADA, it should be taken into consideration that this operation is time consuming, especially in the heterogeneous and high-risk cohort of AADA patients, as was evident in the present as well as comparable studies (8). Although, non-David alternatives such as the CVR might save operation time, drawbacks of non-valve sparing techniques, such as subsequent lifelong anticoagulation in young patients requiring mechanical valve replacement with concomitant risk for thromboembolic and bleeding complications, need to be taken into consideration. However, in selected cases with critical preoperative status the patient might indeed benefit from a fast and less complex approach. Hence, as outlined in the presented treatment algorithm—we advocate for a non-dogmatic treatment strategy, with bailout approaches for the above mentioned cases.

On the other hand, Bentall procedure using composite graft with valve prosthesis may be an alternative option. Yang et al. compared surgical outcome in patients underwent David procedure (*n* = 40) and Bentall procedure (*n* = 95) for AADA (22). The patient background in both groups was heterogeneous as in our present study, but operative mortality was 3% in the David group and 13% in the Bentall group and 10-year Kaplan-Meier survival was 98% in the David group and 57% in the Bentall group. Although they concluded that both the David and Bentall procedures are appropriate surgical approach for aortic root replacement in selected patients with AADA, the late survival of their study may suggest that the David procedure may be superior to the Bentall procedure, if patients survived during perioperative period.

Overall 30-day mortality in our series was approximately 20%, which is comparable to recent reports for the surgical treatment of AADA independent from the employed surgical technique (1, 2, 8, 15). Thirty-day mortality for patients undergoing the *David procedure* was 17.9% and 5-year mortality was 28.6%, with no differences as compared to the non-David group. In addition, early and mid-term aortic valve function after the *David procedure* was excellent despite the AADA setting, and with no valve-related reoperations in the David-group. This is comparable to other recent studies in which the *David procedure* was employed for ADAA treatment (10–15), although—due to the employed treatment strategy with the *David procedure* as first-choice procedure—the proportion of patients receiving a valve-sparing aortic root replacement was much higher than in any other series.

Nonetheless, certain limitations of this study must be acknowledged as this study represents only a small series, in which surgical treatment strategy did not follow randomization but individual judgment. Furthermore, patients undergoing the *David procedure* are inevitably preselected as a valve-sparing technique requires impaired aortic valve cusps, which may correlate to reduced morbidity. Hence, further randomized and long-term studies are warranted to elucidate the benefits of a valve sparing technique over other procedures for the treatment of AADA.

## Conclusion

This small series demonstrates the safety and feasibility of the *David procedure* for surgical treatment of AADA in terms of 30-day and mid-term mortality, as well as valve function after valve-sparing aortic root replacement. Hence, with adequate patient selection criteria the *David procedure* should be considered as a valid first choice treatment strategy for AADA patients despite increased technical complexity of the operation itself.

## Data Availability

The datasets generated for this study are available on reasonable request to the corresponding author.

## Ethics Statement

This study was approved by the ethics committee of the medical faculty of the Heinrich Heine University, complying with the principles outlined in the Declaration of Helsinki. Written informed consent for participation was not required for this study in accordance with the national legislation and the institutional requirements.

## Author Contributions

HA and HK: manuscript description. PA and AL: supervising and revision of the manuscript. PR: echocardiographic examination. CP and GP: data collection and primary analysis. HK: data interpretation.

### Conflict of Interest Statement

The authors declare that the research was conducted in the absence of any commercial or financial relationships that could be construed as a potential conflict of interest.
